# BMI-Stratified Risk of Cesarean Delivery Following Labor Induction: A Robson Classification-Based Cohort Study with Predictive Modeling

**DOI:** 10.3390/jcm15103603

**Published:** 2026-05-08

**Authors:** Sait Erbey, Gizem Aktemur, Mehmet Alican Sapmaz, Ömer Osman Eroğlu, Murat Polat, Bilge Erbey, Betül Tokgöz Çakır, Nazan Vanlı Tonyalı

**Affiliations:** 1 Department of Obstetrics and Gynecology, Ankara Etlik City Hospital, 06170 Ankara, Turkey; 2 Department of Perinatology, Ankara Etlik City Hospital, 06170 Ankara, Turkey; 3 Independent Obstetrics and Gynecology Specialist, 06800 Ankara, Turkey

**Keywords:** body mass index, labor induction, cesarean delivery, Robson classification, risk stratification

## Abstract

**Objectives:** Maternal obesity is associated with adverse labor induction outcomes, but real-world data stratified by the full WHO body mass index (BMI) classification and adjusted for parity remain limited. We evaluated BMI-stratified induction outcomes using the Robson Ten-Group Classification System and developed a combined multivariable model integrating BMI, Bishop score, and parity for pre-induction risk stratification. **Methods:** This single-center retrospective cohort study included 501 singleton term pregnancies undergoing labor induction in Ankara, Turkey (March–August 2023), stratified by five WHO BMI categories. The primary outcome was cesarean delivery. Analyses included Cochran–Armitage trend tests, multivariable logistic regression, and ROC analysis with DeLong comparison and bootstrap optimism-corrected internal validation. The study followed STROBE guidelines. **Results:** The overall cesarean rate was 30.3%, rising from 17.1% (normal weight) to 52.2% (Obese class III; Cochran–Armitage Z = 6.099, *p* < 0.001), with consistent trends across Robson Groups 2 and 4 (both *p* < 0.001). Failed induction increased from 2.9% to 15.2% (*p* < 0.001). The cervical ripening requirement rose from 32.4% to 60.9% (*p* = 0.003). BMI (adjusted odds ratio [aOR] = 1.489, 95% confidence interval [CI] 1.254–1.767) and Bishop score (aOR = 0.807, 95% CI 0.718–0.906) were independent predictors; nulliparity showed a non-significant trend (*p* = 0.090). The combined model (BMI + Bishop + nulliparity) achieved an apparent AUC of 0.715 (optimism-corrected 0.709), outperforming both Bishop score alone (DeLong *p* = 0.010) and BMI alone (*p* = 0.012). Calibration was adequate (Hosmer–Lemeshow *p* = 0.632). **Conclusions:** Higher BMI independently predicts increased cesarean risk following labor induction across parity subgroups. The combined multivariable model provides practical bedside pre-induction risk stratification superior to either individual predictor, though external validation is warranted before widespread clinical implementation.

## 1. Introduction

Global estimates indicate that overweight and obesity have reached epidemic proportions, affecting a substantial proportion of women of reproductive age worldwide [1]. According to the Turkey Health Survey 2022 (Turkish Statistical Institute), approximately 39% of adult women in Turkey are obese, and more than half are overweight or obese, with rising prevalence across successive age cohorts. Maternal obesity is associated with an increased risk of gestational diabetes mellitus, preeclampsia, fetal macrosomia, and post-term pregnancy, all of which may increase the need for labor induction [2,3].

Labor induction is one of the most commonly performed obstetric interventions, accounting for approximately 20–25% of all deliveries in high-income countries, with recent reports from the United States indicating rates as high as one in three singleton births [2]. This proportion is considerably higher among obese women due to the increased prevalence of medical and obstetric indications. Despite its widespread use, induction of labor in obese women remains clinically challenging, with higher rates of failed induction, prolonged labor, and cesarean delivery reported in the literature [2,4,5]. Turkey’s national cesarean delivery rate exceeds 52% (Turkish Statistical Institute, 2022), the highest among Organisation for Economic Co-operation and Development (OECD) countries, making obesity-related induction outcomes a pressing national health priority.

Although previous studies and meta-analyses have demonstrated an association between maternal obesity and adverse labor induction outcomes, the extent to which these effects vary across BMI categories remains incompletely understood. In particular, real-world data stratified according to WHO BMI classification—covering the full range from normal weight to morbid obesity—are limited, especially outside Western European and North American populations. Furthermore, application of the Robson classification to disentangle the effect of parity on BMI-stratified induction outcomes has been underutilized in this context [6,7]. The ARRIVE (A Randomized Trial of Induction Versus Expectant Management) trial demonstrated that elective induction at 39 weeks reduced cesarean delivery among low-risk nulliparous women, with consistent findings across BMI strata, but the generalizability of these results to morbidly obese women in routine practice remains debated [8].

Several biologically plausible mechanisms have been proposed to explain the adverse effects of obesity on induction success. These include impaired myometrial responsiveness to oxytocin, delayed cervical ripening, mechanical narrowing of the birth canal secondary to excess adipose tissue, and an altered pro-inflammatory cytokine profile. Existing systematic reviews estimate that obesity approximately doubles the risk of failed induction, with pooled odds ratios around 1.82 for cesarean birth following labor induction [9,10,11].

The current study addresses this clinical gap by examining BMI-stratified induction outcomes in a real-world Turkish obstetric cohort. To our knowledge, this is one of the few studies to simultaneously incorporate the Robson classification for parity-stratified subgroup analysis, Institute of Medicine (IOM) gestational weight gain categorization, formal epidural confounding assessment, and a combined multivariable predictive model with bootstrap-based internal validation in a single induction cohort. The specific objectives of this study were threefold: (1) to quantify the dose–response relationship between admission BMI and cesarean delivery following labor induction across the full WHO BMI spectrum (normal weight to class III obesity) in a real-world Turkish tertiary-care induction cohort; (2) to disentangle the contribution of parity from that of maternal adiposity by applying the Robson Ten-Group Classification System (Groups 2 and 4); and (3) to develop and internally validate a parsimonious bedside risk-stratification model combining BMI, Bishop score, and parity, and to benchmark its discriminative performance against each predictor used alone. Correspondingly, we hypothesized that (i) BMI would independently predict cesarean delivery after adjustment for parity and Bishop score, (ii) the BMI–cesarean association would persist within both Robson Groups 2 and 4, and (iii) the combined multivariable model would achieve significantly higher discrimination than either single-predictor model. This integrated approach, alongside a focus on practical risk stratification tools applicable at the bedside, represents the principal novelty of this work.

## 2. Materials and Methods

### 2.1. Study Design and Setting

This single-center retrospective cohort study was conducted at the Obstetrics and Gynecology Clinic of Ankara Etlik City Hospital, a tertiary referral center in Ankara, Turkey. Ankara Etlik City Hospital is a high-volume tertiary referral center serving both routine obstetric admissions from the surrounding metropolitan area and high-risk pregnancies referred from primary and secondary care facilities across the Central Anatolia region. The induction cohort therefore reflects a mixed-risk obstetric population, with a higher proportion of medical and obstetric induction indications than would be expected in primary care settings; this contextual feature should be considered when generalizing the findings to lower-acuity settings. The study included consecutive singleton pregnancies that underwent labor induction between March 2023 and August 2023. As a pragmatic observational study, all consecutive eligible patients during the study period were included, yielding a sample of 501 women. Following ethics committee approval on 6 September 2023, retrospective data extraction from the institutional electronic medical record system was conducted between September 2023 and June 2024, and statistical analyses were performed between July 2024 and February 2025. All data extraction and analytic work were carried out at Ankara Etlik City Hospital, Ankara, Turkey. A post hoc power analysis (detailed in Section 2.7) demonstrated adequate statistical power for the primary comparison. The study was reported in accordance with the Strengthening the Reporting of Observational Studies in Epidemiology (STROBE) guidelines [12].

### 2.2. Participants

Inclusion criteria were: singleton viable pregnancy, cephalic presentation, gestational age ≥ 37 weeks, and no prior cesarean delivery or other major uterine surgery (e.g., myomectomy). Exclusion criteria included multiple gestation, congenital uterine anomaly, placenta previa, major fetal anomaly, pre-existing chronic hypertension, pregestational (type 1 or type 2) diabetes mellitus, and maternal age below 18 years. Of note, women with diet-controlled gestational diabetes mellitus (GDM) were eligible for inclusion and represented 47 patients (9.4% of the cohort), as GDM is a common indication for labor induction and reflects the real-world induction population. Eligibility criteria also permitted inclusion of women with gestational or new-onset hypertensive disorders of pregnancy; however, no patient with a primary induction indication of gestational hypertension or preeclampsia was recorded during the study period, and this comorbidity therefore did not contribute to the analytic cohort. We acknowledge that GDM is associated with fetal macrosomia and may increase cesarean risk through cephalopelvic disproportion, and that its prevalence and clinical impact may vary with maternal BMI. In our cohort, the distribution of GDM across the five BMI categories was 15, 14, 9, 7, and 2 women from normal weight through Obese class III, respectively. Subgroup stratification by GDM status was not undertaken as a prespecified analysis because (i) the study was designed to evaluate the overall BMI–induction relationship in a real-world cohort rather than within disease-specific subgroups, and (ii) further stratification beyond the five WHO BMI categories and two Robson groups would have produced cells with limited statistical precision (e.g., only 2 GDM cases in the Obese III stratum). To address potential residual confounding, induction indication—which captures GDM as a discrete category—was included as a covariate in the prespecified sensitivity analysis (Section 2.7), which did not materially alter the BMI effect estimate (likelihood ratio χ^2^ = 2.24, *p* = 0.896). Pregnancies complicated by pre-existing chronic hypertension and pregestational diabetes mellitus were excluded a priori, as listed above. Of the initially screened 542 inductions during the study period, 41 were excluded (14 with prior cesarean delivery or major uterine surgery, 11 with multiple gestation, 8 with major fetal anomaly, 5 with pregestational diabetes, and 3 with placenta previa), yielding the final analytic cohort of 501 women with complete data for all primary analyses.

### 2.3. Exposure: Body Mass Index Assessment

BMI was calculated using maternal weight measured at the time of hospital admission for labor induction—that is, the index admission at term gestation (≥37 weeks) immediately preceding the induction procedure, rather than at any earlier antenatal contact, outpatient visit, or admission for unrelated obstetric care. To avoid ambiguity in this referral-based cohort, where patients may have had earlier antenatal contacts at varying gestational ages, weight and height used to compute the exposure variable were taken exclusively from this index induction admission. Maternal weight was measured on a calibrated standing scale by trained obstetric nursing staff according to standard institutional protocol, and height was measured at the same admission. BMI was computed as admission weight (kg) divided by height squared (m^2^) and categorized according to the WHO classification into five groups: normal weight (18.5–24.9 kg/m^2^), overweight (25–29.9 kg/m^2^), Obese class I (30–34.9 kg/m^2^), Obese class II (35–39.9 kg/m^2^), and Obese class III (≥40 kg/m^2^). No patients in the underweight category (<18.5 kg/m^2^) were identified; therefore, analyses were conducted across the five aforementioned groups.

Important methodological consideration regarding BMI classification: Because pre-pregnancy BMI data were not systematically available for the entire cohort, admission BMI was used as the primary exposure variable for all main analyses. Admission BMI reflects combined baseline adiposity and gestational weight gain (GWG) at term (approximately 8–12 kg in the typical range). Consequently, strict alignment with the standard WHO pre-pregnancy BMI-based definition of “maternal obesity” is not possible; the exposure should be interpreted as *adiposity at term* rather than pre-pregnancy BMI. Self-reported pre-pregnancy weight was recorded at the first antenatal visit at our institution, which most commonly occurred between approximately 6 and 14 weeks of gestation, in line with standard Turkish antenatal care practice; a subset of patients referred from primary or secondary care first presented later in pregnancy. Pre-pregnancy weight was obtained by patient recall in all 501 women (100% of the cohort), as objective pre-conception weight measurements were not routinely available in the institutional electronic medical record system. We acknowledge that self-reported pre-pregnancy weight is subject to recall bias and systematic underestimation; this limitation is explicitly addressed in Section 4.2. Self-reported pre-pregnancy weight was used solely for the secondary IOM gestational weight gain analysis and was not used for the primary BMI exposure variable, which was based on directly measured admission weight at the time of induction. GWG was calculated as the difference between admission weight and self-reported pre-pregnancy weight and classified according to the 2009 IOM guidelines [13] into three groups: below recommended, within recommended, and excessive. IOM categories were assigned based on self-reported pre-pregnancy BMI, whereas the primary exposure variable in all other analyses was admission BMI. This distinction should be noted when interpreting the IOM analysis.

### 2.4. Robson Ten-Group Classification

The Robson Ten-Group Classification System was applied to stratify patients by parity and other obstetric characteristics [6,7]. Because this cohort comprised exclusively induced singleton term pregnancies without a uterine scar, all patients were categorized into either Robson Group 2 (nulliparous women undergoing induction) or Robson Group 4 (multiparous women without a previous uterine scar undergoing induction), allowing parity-adjusted comparison of cesarean rates across BMI categories.

### 2.5. Outcomes

The primary outcome was mode of delivery (vaginal delivery vs. cesarean section). Secondary outcomes included Bishop score at the time of induction, total induction-to-delivery interval (hours), duration of the latent phase (hours), duration of the active phase (hours), use of cervical ripening agents, use of epidural analgesia during labor, birth weight (grams), Apgar scores at 1 and 5 min, neonatal intensive care unit (NICU) admission, and distribution of cesarean delivery indications. Failed induction was defined according to the American College of Obstetricians and Gynecologists (ACOG) criteria [14] as inability to achieve the active phase of labor (cervical dilation ≥ 6 cm) despite adequate uterine contractions (≥3 contractions per 10 min, each lasting ≥40 s) following a minimum of 12 h of oxytocin infusion after cervical ripening, or following dinoprostone/Foley catheter ripening when applicable. A Bishop score threshold of <4 was used to define an unfavorable cervix, consistent with published evidence and clinical practice at our institution [15,16,17,18].

### 2.6. Induction Protocol

Induction agents included intravenous oxytocin (low-dose incremental protocol, starting at 1–2 mU/min, increased by 1–2 mU/min every 30–40 min to a maximum of 20 mU/min), vaginal dinoprostone 10 mg controlled-release insert (Propess^®^, Ferring Pharmaceuticals, Saint-Prex, Switzerland), and transcervical Foley catheter balloon (30 mL) [19,20,21]. Agent selection was based on cervical status at admission: women with Bishop score < 4 received cervical ripening prior to oxytocin augmentation, while those with Bishop score ≥ 4 proceeded directly to oxytocin induction. Detailed oxytocin dosing data (total dose administered, duration at maximum rate) were not systematically retrievable from the electronic records and could not be included in the analyses.

### 2.7. Statistical Analysis

All statistical analyses were performed using IBM SPSS Statistics (version 27; IBM Corp., Armonk, NY, USA) and R (version 4.3.0; R Foundation for Statistical Computing, Vienna, Austria). Continuous variables are presented as mean ± standard deviation (SD) or median with interquartile range (IQR) depending on distribution; categorical variables as count and percentage (%). Ordinal variables (Apgar scores, Bishop score) are reported as median (IQR) given their non-interval nature. Between-group comparisons for continuous variables were performed using the Kruskal–Wallis test, as the Shapiro–Wilk test confirmed non-normal distribution of key variables. The chi-squared test was used for categorical comparisons. Associations between BMI and continuous variables were assessed using Spearman rank correlation. The Cochran–Armitage trend test was applied to evaluate linear trends in cesarean delivery rates and failed induction rates across ordered BMI categories.

Primary outcome analyses were: (i) the Cochran–Armitage trend test for cesarean delivery across BMI categories overall and within Robson Groups 2 and 4; (ii) multivariable logistic regression identifying independent predictors of cesarean delivery. Variable selection for the multivariable model was prespecified based on clinical relevance and prior literature: BMI as the primary exposure, Bishop score as the strongest established predictor of induction outcome, and nulliparity as a major effect-modifier of cesarean risk. To preserve model parsimony and avoid overfitting given the 152 cesarean events (events-per-variable ≈ 50), the model was restricted to these three core predictors rather than including all available baseline covariates. All other analyses were considered secondary/exploratory. Given the pre-specified primary outcome focus, no formal adjustment for multiple comparisons was applied; reported *p*-values for secondary outcomes should therefore be interpreted as hypothesis-generating.

Binary logistic regression analysis was performed with mode of delivery (cesarean vs. vaginal) as the dependent variable. BMI category was entered as an ordinal variable (1 = normal weight, 2 = overweight, 3 = Obese I, 4 = Obese II, 5 = Obese III), with a linear trend assumption across levels; nulliparity and admission Bishop score were included as additional covariates. Nulliparity was retained in the final model a priori based on its established clinical relevance to labor outcomes, its documented interaction with BMI in influencing induction outcomes, and its significant distribution across BMI groups (*p* = 0.043), regardless of its statistical significance in the multivariable model. Results are reported as adjusted odds ratios (aORs) with 95% CIs. A sensitivity analysis was performed by additionally including induction indication as a covariate, with model fit compared using the likelihood ratio test. A second prespecified sensitivity analysis substituted self-reported pre-pregnancy BMI for admission BMI as the exposure variable, to assess the robustness of the BMI–cesarean association to the choice of BMI measurement timing. Model calibration was assessed using the Hosmer–Lemeshow goodness-of-fit test across deciles of predicted probability.

Receiver operating characteristic (ROC) curve analysis was performed to evaluate the predictive performance of BMI, Bishop score, and a combined multivariable logistic model incorporating BMI category, Bishop score, and nulliparity. For the single-predictor ROC analyses, BMI was entered as a continuous variable (kg/m^2^) and Bishop score as a continuous ordinal variable, while the combined model used the same categorical BMI specification as the multivariable logistic regression to ensure methodological consistency between effect estimation and discrimination assessment. Pairwise comparisons of AUC values were performed using the DeLong method with 95% CIs estimated by bootstrap resampling (2000 iterations). Because the combined model AUC was derived from the same dataset used for model fitting, we additionally performed bootstrap optimism-corrected (Harrell’s method) internal validation with 2000 resamples to provide a more realistic estimate of out-of-sample performance [22]. A post hoc power analysis comparing the Obese III and normal weight groups (52.2% vs. 17.1%) demonstrated a power of 99.3% (z-test, α = 0.05, Cohen’s h = 0.761). Statistical significance was defined as *p* < 0.05.

### 2.8. Measures to Address Potential Sources of Bias

Several methodological strategies were applied to limit bias and enhance internal validity. *Selection bias* was minimized by enrolling all consecutive eligible inductions during the study period, with prespecified exclusion criteria (Section 2.2). *Information bias* was reduced by extracting all variables directly from the institutional electronic medical record using standardized definitions, with complete outcome ascertainment (no loss to follow-up). *Measurement bias* in the primary exposure was limited by using directly measured (rather than self-reported) admission weight and height (Section 2.3). *Confounding* was addressed through Robson Group 2 versus Group 4 stratification, multivariable logistic regression adjusting for nulliparity and Bishop score, and a sensitivity analysis additionally adjusting for induction indication. *Misclassification* of the primary exposure (admission vs. pre-pregnancy BMI) and *recall bias* affecting self-reported pre-pregnancy weight—the latter restricted to the secondary IOM analysis—are acknowledged transparently in Section 2.3 and Section 4.2. *Optimism bias* in apparent model performance was addressed using Harrell’s bootstrap optimism-corrected internal validation (2000 resamples; Section 2.7).

### 2.9. Ethical Approval

The study was conducted after obtaining approval from the Clinical Research Ethics Committee of Ankara Etlik City Hospital (date: 6 September 2023, decision no: AEŞH-EK1-2023-400). All procedures were conducted in accordance with ethical guidelines and the principles set forth in the Declaration of Helsinki. Because of the retrospective observational design using routinely collected de-identified clinical data, the requirement for individual informed consent was waived by the ethics committee.

## 3. Results

**Figure 1:**
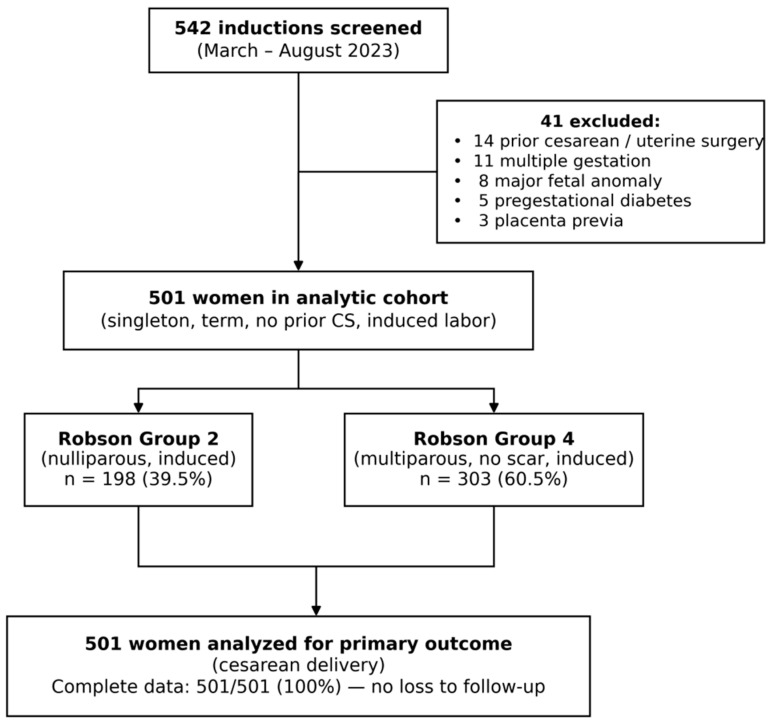
Study flow diagram according to the STROBE statement. Of 542 induced labors screened during the study period (March–August 2023), 41 were excluded based on prespecified criteria, yielding the final analytic cohort of 501 women. Patients were stratified into Robson Group 2 (nulliparous undergoing induction; *n* = 198) and Robson Group 4 (multiparous without uterine scar undergoing induction; *n* = 303). Complete outcome data were available for all 501 women, with no loss to follow-up. CS: cesarean section.

A total of 501 women were included in the analytic cohort (Figure 1, Table 1). The mean maternal age was 27.8 ± 5.4 years, the mean admission BMI was 32.1 ± 6.4 kg/m^2^, and the mean gestational age at induction was 40.1 ± 1.5 weeks. The largest group was overweight (*n* = 145, 28.9%), followed by Obese I (*n* = 130, 25.9%), normal weight (*n* = 105, 20.9%), Obese II (*n* = 75, 15.0%), and Obese III (*n* = 46, 9.2%). The overall nulliparity rate was 39.5% (*n* = 198), which differed significantly across BMI groups (*p* = 0.043), motivating the Robson-stratified analysis. The most common indications for induction were post-term pregnancy (33.5%, *n* = 168), abnormal non-stress test or biophysical profile (21.0%, *n* = 105), and prelabor rupture of membranes (19.0%, *n* = 95). The admission Bishop score decreased significantly with increasing BMI (normal: median 5 (IQR 4–6) vs. Obese III: median 3 (IQR 2–4); Kruskal–Wallis H = 42.48, *p* < 0.001; Spearman r_s_ = −0.276, *p* < 0.001).

**Figure 2:**
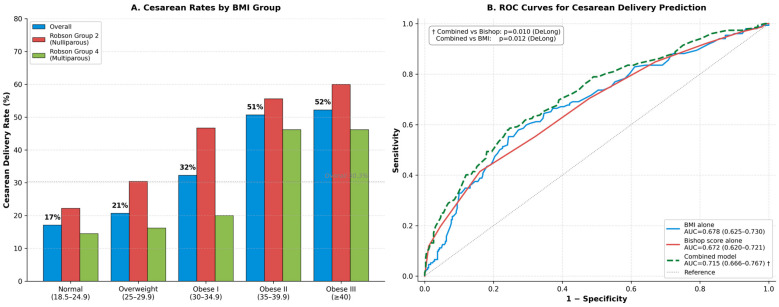
(**A**) Cesarean delivery rates by BMI group, overall and stratified by Robson classification (Group 2: nulliparous; Group 4: multiparous). The dashed reference line denotes the overall cesarean rate (30.3%). (**B**) Receiver operating characteristic (ROC) curves for prediction of cesarean delivery following labor induction. AUC values with 95% CIs: BMI alone = 0.678 (0.625–0.730); Bishop score alone = 0.672 (0.620–0.721); combined model (BMI + Bishop + nulliparity) = 0.715 (0.666–0.767). DeLong test: combined vs. Bishop alone Z = 2.593, *p* = 0.010; combined vs. BMI alone Z = 2.516, *p* = 0.012.

The overall cesarean delivery rate was 30.3% (*n* = 152), increasing progressively with BMI: 17.1% in the normal group, 20.7% in overweight, 32.3% in Obese I, 50.7% in Obese II, and 52.2% in the Obese III group (Cochran–Armitage Z = 6.099, *p* < 0.001). When analyzed by Robson classification, the BMI-associated trend in cesarean rates was consistently observed in both parity subgroups. In Robson Group 2 (nulliparous, *n* = 198), cesarean rates increased from 22.2% in normal-weight to 60.0% in Obese III women (Z = 3.688, *p* < 0.001). In Robson Group 4 (multiparous, *n* = 303), rates increased from 14.5% to 46.2% (Z = 4.448, *p* < 0.001). The overall cesarean rate was significantly higher in Group 2 compared to Group 4 (41.4% vs. 23.1%; χ^2^ = 18.14, *p* < 0.001). The most common indications for cesarean delivery were non-reassuring fetal heart rate patterns (34.9%, *n* = 53), prolonged active phase/arrest (21.1%, *n* = 32), and failed induction (19.1%, *n* = 29). Mode of delivery and Robson-stratified rates are presented in Table 2 and illustrated in Figure 2A.

To explore whether the progressive rise in cesarean delivery was accompanied by a shift in the underlying clinical reasons, the distribution of indications was analyzed by BMI group. The distribution of cesarean delivery indications did not differ significantly across BMI groups (χ^2^ = 6.56, df = 16, *p* = 0.981; confirmed by Monte Carlo simulation given low expected cell counts, *p* = 0.982). However, failed induction as a specific indication showed a statistically significant linear trend across BMI categories (Cochran–Armitage Z = 3.570, *p* < 0.001), rising from 2.9% in the normal group to 15.2% in the Obese III group when expressed as a proportion of all women in each BMI category. Cesarean indication data are presented in Table 3.

BMI correlated significantly and positively with total induction duration (r_s_ = +0.337, *p* < 0.001; Kruskal–Wallis H = 62.28, *p* < 0.001), latent phase duration (r_s_ = +0.310, *p* < 0.001; H = 51.49, *p* < 0.001), and active phase duration (r_s_ = +0.381, *p* < 0.001; H = 80.55, *p* < 0.001). Latent phase duration increased from a mean of 3.5 ± 2.1 h in the normal group to 6.0 ± 3.1 h in the Obese III group. Women who delivered vaginally had a mean total induction duration of 7.79 h, compared with 9.85 h in those who underwent cesarean delivery (Mann–Whitney *p* < 0.001). Cervical ripening requirement increased progressively with BMI, from 32.4% in the normal group to 60.9% in Obese III (χ^2^ = 15.90, *p* = 0.003), with a corresponding shift from oxytocin-only management (67.6% → 39.1%) toward mechanical and pharmacological ripening. Epidural analgesia was used in 31 women (6.2%), with no significant difference across BMI groups (χ^2^ = 1.13, *p* = 0.890); it was more frequent among nulliparous (Robson Group 2) than multiparous (Robson Group 4) women (10.6% vs. 3.3%; χ^2^ = 9.79, *p* = 0.002). The Spearman correlation between BMI and active phase duration was virtually unchanged when epidural recipients were excluded (r_s_ = 0.392 vs. 0.381), suggesting minimal confounding by differential epidural use. Labor duration, induction agent distribution, and epidural data are presented in Table 4.

In multivariable logistic regression, BMI category (aOR = 1.489 per one-level increase, 95% CI 1.254–1.767, *p* < 0.001) and admission Bishop score (aOR = 0.807 per unit increase, 95% CI 0.718–0.906, *p* < 0.001) were independent predictors of cesarean delivery. Nulliparity showed a trend toward association (aOR = 1.481, 95% CI 0.940–2.332, *p* = 0.090) but did not reach statistical significance. The Nagelkerke R^2^ of the full model was 0.174, and the model showed adequate calibration (Hosmer–Lemeshow χ^2^ = 6.14, df = 8, *p* = 0.632). A sensitivity analysis incorporating induction indication as a covariate did not meaningfully alter any estimate (likelihood ratio test χ^2^ = 2.24, *p* = 0.896). Regression results are presented in Table 5.

In a prespecified sensitivity analysis substituting self-reported pre-pregnancy BMI for admission BMI as the exposure variable, the association with cesarean delivery remained essentially unchanged. The adjusted odds ratio for pre-pregnancy BMI category was 1.461 (95% CI 1.227–1.739, *p* < 0.001), with Bishop score (aOR = 0.801, 95% CI 0.714–0.899, *p* < 0.001) and nulliparity (aOR = 1.442, 95% CI 0.918–2.264, *p* = 0.112) showing comparable estimates to the primary model. The model AUC was 0.711 (compared with 0.715 in the primary analysis), and the Cochran–Armitage trend test for cesarean delivery across pre-pregnancy BMI categories was similarly robust (Z = 5.842, *p* < 0.001). Of note, 11.0% of women (*n* = 55) were classified into a different (one category higher) BMI stratum when using pre-pregnancy rather than admission BMI, reflecting the typical 2–3 kg/m^2^ shift introduced by gestational weight gain. These findings indicate that the BMI–cesarean association is not meaningfully altered by the choice of BMI measurement timing (full results, Table 5B).

Receiver operating characteristic (ROC) curve analysis demonstrated apparent AUC values of 0.678 (95% CI 0.625–0.730) for BMI alone, 0.672 (95% CI 0.620–0.721) for the Bishop score alone, and 0.715 (95% CI 0.666–0.767) for the combined multivariable model incorporating BMI, Bishop score, and nulliparity (Figure 2B). The combined model demonstrated significantly higher discrimination than both the Bishop score alone (DeLong Z = 2.593, *p* = 0.010) and BMI alone (DeLong Z = 2.516, *p* = 0.012). To assess the robustness and potential optimism of these predictive models, bootstrap optimism-corrected internal validation was performed (Harrell’s method, 2000 resamples). Across all three models, the magnitude of optimism was negligible (≤0.006 AUC units), indicating that apparent AUC estimates closely approximate expected out-of-sample performance within this cohort. The optimism-corrected AUC for the combined model was 0.709, while both single-predictor models showed near-zero or slightly negative optimism values (BMI alone: −0.001; Bishop alone: ≈0.000), which is characteristic of parsimonious models with few parameters and limited overfitting potential. Summary statistics for apparent AUC, optimism estimates, optimism-corrected AUC, and DeLong comparisons are presented in Table 6.

As a secondary analysis, gestational weight gain (mean ± SD) by BMI category is shown in Table 1; mean GWG was highest among normal-weight women and decreased with increasing baseline BMI. When classified according to IOM 2009 criteria [13] (applied based on self-reported pre-pregnancy BMI), the distribution of below/within/excessive categories was 30.5%/67.6%/1.9% for normal-weight, 32.4%/56.6%/11.0% for overweight, 19.2%/47.7%/33.1% for Obese I, 12.0%/42.7%/45.3% for Obese II, and 21.7%/37.0%/41.3% for Obese III women (χ^2^ = 79.33, *p* < 0.001). Excessive GWG was thus most prevalent in the Obese I–III groups, while normal-weight women most frequently gained within the recommended range. Importantly, cesarean delivery rates did not differ significantly by IOM category (below IOM: 30.9%; within IOM: 28.8%; above IOM: 33.3%; χ^2^ = 0.802, *p* = 0.670), suggesting that baseline adiposity rather than gestational weight gain magnitude drives induction outcomes.

## 4. Discussion

Our findings extend the existing literature in three principal ways. First, we demonstrate a dose–response gradient between admission BMI and cesarean delivery that persists after rigorous adjustment for parity through Robson stratification—an analytic framework rarely applied in the BMI–induction literature—suggesting that the BMI effect is not simply mediated by the higher nulliparity prevalence in obese cohorts but represents an independent biological signal. The robustness of this gradient across both nulliparous and multiparous women supports a unifying mechanistic framework rather than a parity-specific phenomenon, consistent with meta-analytic estimates indicating approximately 1.82-fold cesarean odds in obese induced women [9,10,23]. Second, the overall cesarean rate in our induction cohort, although markedly lower than the Turkish national rate of >52% (Turkish Statistical Institute, 2022), should be interpreted in context: the national figure encompasses repeat cesareans, non-induced deliveries, and elective procedures across all parity and risk strata, whereas our cohort reflects an exclusively term, scar-free induction population. Aligning our findings with international standardized induction cohorts [24] reinforces the external coherence of the BMI–cesarean dose–response in this clinically homogeneous setting and underscores the value of induction-specific—rather than population-wide—benchmarks for obesity-related obstetric risk.

Cervical ripening requirements increased progressively from 32.4% to 60.9% across BMI groups, consistent with evidence that obesity impairs cervical maturation through altered prostaglandin metabolism and pro-inflammatory cytokine profiles [25]. The corresponding shift from oxytocin-only to mechanical/pharmacological ripening in higher BMI groups has direct implications for resource planning and counseling [19,20].

BMI correlated significantly with total (r_s_ = +0.337), latent (r_s_ = +0.310), and active phase duration (r_s_ = +0.381; all *p* < 0.001). The latent phase prolongation (3.5 → 6.0 h) likely reflects impaired myometrial oxytocin receptor signaling and mechanical adipose-tissue effects on cervical effacement. Obesity-related hormonal alterations—including altered progesterone/estrogen ratios, reduced gap junction density, and diminished prostaglandin responsiveness—further impair myometrial contractility independent of mechanical factors [25], consistent with findings from Ellekjaer et al. [26] and Polónia Valente et al. [27].

Epidural analgesia use (6.2%) did not differ across BMI groups (*p* = 0.890), and the BMI–active phase correlation was virtually unchanged after excluding epidural recipients (r_s_ = 0.392 vs. 0.381), supporting the interpretation that labor prolongation reflects direct adiposity effects on myometrial and cervical function rather than differential epidural use [27].

The Robson analysis underscores the compounding risk of nulliparity: Group 2 had a 41.4% cesarean rate vs. 23.1% in Group 4 (χ^2^ = 18.14, *p* < 0.001), with Obese III nulliparous women reaching 60.0% [6,7]. The absolute increase in cesarean risk was greater in Group 2 (+37.8 percentage points, from 22.2% to 60.0%) than in Group 4 (+31.7 percentage points, from 14.5% to 46.2%), suggesting that nulliparous women are particularly susceptible to the adverse effects of obesity on induction outcomes, likely due to the absence of a previously favorable cervical remodeling history [26]. Although nulliparity was not a statistically significant independent predictor in the multivariable model (aOR = 1.481, *p* = 0.090), its clinically relevant interaction with BMI supports parity-adjusted risk frameworks [23].

The progressive rise in failed induction with increasing BMI observed in our cohort indicates that maternal adiposity impairs not only intrapartum labor efficiency but also the upstream process of cervical ripening and labor initiation itself. This observation is consistent with prior reports by Drummond et al. [11] and Hamm et al. [24], and supports a multi-mechanism explanation that integrates pharmacokinetic, pharmacodynamic, and physiological dimensions. At the pharmacokinetic level, increased adipose volume of distribution may dilute prostaglandin and oxytocin concentrations at the target tissue, attenuating their effective potency [25]. At the pharmacodynamic level, downregulation and reduced sensitivity of myometrial oxytocin receptors in obesity have been demonstrated experimentally, plausibly contributing to the higher oxytocin requirements and longer induction-to-delivery intervals reported in obese cohorts [25,26]. Mechanically, increased pelvic soft-tissue thickness and altered uterine contractility further compound these effects. Recognizing failed induction as a multi-factorial phenotype—rather than a unitary endpoint of inadequate uterine response—has direct clinical implications: it argues for individualized, BMI-aware induction protocols, including consideration of higher oxytocin titration ceilings, sequential rather than parallel ripening strategies, and earlier reassessment of induction trajectory in women at the upper end of the BMI spectrum.

These findings warrant consideration within the broader debate on optimal timing and management of induction in obese women. The ARRIVE trial demonstrated that elective induction at 39 weeks reduced cesarean delivery among low-risk nulliparous women, and post hoc subgroup analyses suggested consistent benefit across BMI categories [8]. The Krogh et al. [23] systematic review and meta-analysis likewise supported induction over expectant management in obesity. However, our data indicate that the absolute cesarean risk in class II–III obesity remains substantial (>50%), underscoring the need for obesity-tailored induction protocols and individualized counseling rather than a one-size-fits-all approach. Current ACOG guidance emphasizes individualized labor management [21], and our BMI-stratified data provide quantitative support for such individualization, particularly regarding the selection of ripening agent, duration of induction attempts, and timing thresholds for conversion to cesarean delivery.

The principal contribution of our predictive modeling is conceptual rather than algorithmic. Although the combined model—integrating admission BMI, Bishop score, and nulliparity—achieved moderate discriminative performance (AUC ≈ 0.72) that significantly exceeded either single predictor, its more important demonstration is that no individual variable, however clinically familiar, captures the full pre-induction risk landscape: cervical favorability, maternal adiposity, and parity each contribute non-redundant information to the cesarean prediction problem. This supports a paradigm shift away from the historical reliance on Bishop score alone toward multidimensional, individualized risk stratification at the bedside. Importantly, the bootstrap optimism-corrected estimates indicated minimal overfitting, suggesting that the observed model behavior is unlikely to be an artifact of within-sample optimism; nevertheless, internal validation cannot substitute for external testing. Before clinical implementation, the model requires prospective validation in geographically and demographically distinct populations, calibration assessment in low-resource and high-volume settings, and translation into a usable decision-support format—ideally with patient-acceptable probability thresholds defined through shared decision-making research rather than purely statistical optimization. From a practical standpoint, the moderate AUC observed (~0.72) reflects a familiar clinical reality: pre-induction risk is multifactorial, and even a well-fitted model cannot deterministically classify individual patients but can usefully inform probability-based counseling. We deliberately do not propose a specific decision threshold (e.g., a probability cut-off above which induction would be discouraged), because the appropriate threshold depends on patient-specific values regarding the relative acceptability of cesarean delivery versus prolonged induction or failed trial of labor—values that vary substantially across individuals and cultural contexts and are best elicited through shared decision-making rather than imposed by an external rule. Until these steps are completed, the model is best viewed as a hypothesis-generating framework that reframes how clinicians should conceptualize pre-induction counseling, rather than as a ready-to-deploy clinical tool.

Although the discrimination of the combined model is modest, its strength lies in its relative simplicity and immediate bedside applicability without the need for additional biomarkers or computational tools.

Birth weight (*p* = 0.019) and 1 min Apgar scores (*p* = 0.016) differed across BMI groups, consistent with prior reports [25]. The Apgar decline represents a modest shift within the normal range (Apgar < 7 rate: *p* = 0.236), without clinically significant neonatal depression. As this difference did not reach statistical significance and was identified within an exploratory secondary analysis without adjustment for multiple comparisons, it should be interpreted as hypothesis-generating rather than as evidence of a clinically meaningful adiposity-related neonatal effect. NICU rates showed a numerical gradient (2.9% → 8.7%) that did not reach statistical significance (*p* = 0.338), likely due to limited power (25 NICU admissions total); this finding therefore should not be interpreted as evidence for or against a true effect. The absence of a GWG–cesarean association (*p* = 0.670) suggests that baseline adiposity, not gestational weight gain, primarily drives induction outcomes.

### 4.1. Clinical Implications

Three clinically actionable points emerge from these data. First, BMI category and admission Bishop score—both readily available at the bedside—provide meaningful risk stratification prior to induction. For every one-category increase in BMI, the odds of cesarean delivery rise by approximately 50% after adjustment for parity and cervical favorability. Second, nulliparous women with class III obesity face an absolute cesarean risk of 60%, with a failed induction rate of 15.2%; this magnitude warrants explicit pre-induction counseling regarding realistic probabilities of vaginal delivery and shared decision-making about timing and agent selection. Third, the progressive rise in cervical ripening requirements (32.4% → 60.9%) indicates that service planning—including allocation of ripening-dedicated beds, pharmacy stocking, and nursing workload estimates—should be calibrated to the BMI distribution of the local induction population.

### 4.2. Strengths and Limitations

Strengths of this study include complete WHO BMI stratification across all five categories, application of the Robson Ten-Group Classification System enabling parity-adjusted analysis, inclusion of IOM GWG data, formal assessment of epidural confounding, DeLong-based ROC comparisons with bootstrap optimism-corrected internal validation, formal calibration assessment (Hosmer–Lemeshow), and a post hoc power of 99.3% (Cohen’s h = 0.761) for the primary comparison.

Several limitations warrant acknowledgment. First, the retrospective single-center design at a Turkish tertiary referral hospital may limit generalizability to other populations and clinical settings; in particular, our findings reflect local induction practices (institutional preferences for ripening-agent selection, oxytocin titration protocols, and timing of cesarean conversion thresholds) that may differ from those of other centers, and findings should be interpreted alongside the institution-specific epidural utilization context discussed below. Second, we used admission BMI rather than pre-pregnancy BMI as the primary exposure variable; this approach captures adiposity at term (combined baseline BMI plus gestational weight gain) rather than strict WHO pre-pregnancy obesity categories, introducing potential misclassification relative to standard definitions. Because admission BMI reflects both baseline adiposity and gestational weight gain, our findings should therefore be interpreted as reflecting term adiposity rather than strictly pre-pregnancy obesity; from a clinical decision-making perspective; however, admission BMI may be more relevant than pre-pregnancy BMI, as it represents the patient’s actual physiological state at the time of induction. The expected direction of any resulting misclassification is non-differential with respect to the cesarean outcome, which under classical measurement-error theory tends to bias effect estimates toward the null; the dose–response gradient we observed therefore likely represents a conservative rather than inflated estimate of the true association. Direct comparisons with literature based strictly on pre-pregnancy BMI should be interpreted with this caveat in mind. To formally assess this concern, we performed a sensitivity analysis substituting self-reported pre-pregnancy BMI for admission BMI; the BMI–cesarean association was essentially unchanged (Section 3, Table 5B), supporting the robustness of our primary findings to the choice of BMI measurement timing. Third, pre-pregnancy weight was self-reported, which is subject to recall bias and systematic underestimation, and this limitation particularly affects the secondary IOM GWG analysis. Fourth, the Obese III group was relatively small (*n* = 46), and although the Cochran–Armitage trend test is appropriate for ordinal BMI categories, subgroup precision in the Obese III stratum is limited. Fifth, the AUC estimates, while supported by bootstrap internal validation, reflect in-sample performance and require external validation in independent cohorts. Sixth, detailed oxytocin dosing data (total dose, duration at maximum infusion rate) were not systematically retrievable from electronic records. Seventh, the low overall epidural use rate (6.2%)—substantially lower than the 50–80% reported in many Western cohorts—reflects well-documented institutional and contextual practice patterns rather than clinical contraindications, and warrants additional explanation for international readers. At our institution, several converging factors account for the low utilization. First, patient demand for intrapartum epidural analgesia is generally low, with the majority of laboring women either preferring unmedicated labor or not actively requesting epidural placement. Second, some obstetricians remain cautious about routine epidural use during induced labor, in part because of long-standing concerns regarding potential prolongation of labor and possible increases in operative or instrumental delivery rates. Third, although a dedicated obstetric anesthesia service is available, the anesthesia team simultaneously covers all obstetric and gynecologic operating rooms in a high-volume tertiary unit; on-call workload, particularly during night shifts, can constrain the practical availability of timely epidural placement for non-urgent intrapartum analgesia. Together, these factors result in a selective rather than routine pattern of epidural utilization. International readers should therefore interpret our labor-duration and induction-trajectory findings within this lower-utilization context, and direct transfer of these estimates to settings where routine epidural availability is the norm (and where epidural-related labor prolongation may itself contribute to longer induction-to-delivery intervals) warrants caution. Eighth, although our multivariable model adjusted for the principal clinical predictors and a sensitivity analysis incorporated induction indication, residual confounding cannot be excluded—particularly with respect to unmeasured variability in induction-protocol implementation (oxytocin titration ceilings, ripening-agent sequencing, and intrapartum decision thresholds) and unrecorded comorbidities, all of which may operate downstream of admission BMI. Ninth, multiple secondary outcomes were assessed without formal adjustment for multiple comparisons; while the primary outcome (cesarean delivery) was prespecified and a priori powered, reported *p*-values for secondary endpoints (induction duration, ripening agent use, neonatal parameters) should be interpreted with appropriate caution as exploratory and hypothesis-generating, not as confirmatory. Finally, we did not assess body fat distribution (visceral vs. subcutaneous adiposity) or long-term neonatal outcomes beyond immediate postnatal parameters.

## 5. Conclusions

Higher admission BMI is associated with reduced cervical favorability, increased cervical ripening requirements, prolonged labor phases, and higher rates of failed induction, and independently predicts greater cesarean delivery risk following labor induction. These associations were robust to multivariable adjustment and consistent across parity subgroups by Robson classification, with Obese III nulliparous women showing the highest risk (60.0% cesarean rate, 15.2% failed induction rate). The combined multivariable model integrating BMI, Bishop score, and nulliparity (apparent AUC = 0.715; bootstrap optimism-corrected AUC = 0.709) demonstrated significantly higher discrimination than either individual predictor (DeLong *p* = 0.010 vs. Bishop alone; *p* = 0.012 vs. BMI alone), supporting its potential use as a bedside tool for pre-induction risk stratification. Because all three components—BMI category, admission Bishop score, and parity—are routinely available at the time of induction decision-making, the model offers a practical, low-cost approach to individualized counseling. This is particularly valuable in women with class II–III obesity, where shared decision-making about induction timing, agent selection, and realistic probabilities of vaginal delivery becomes most relevant. Future prospective multicenter studies with external validation should confirm these findings and assess whether obesity-tailored induction protocols can mitigate the excess cesarean risk identified in this cohort.

## Figures and Tables

**Table 1:** Baseline demographic, clinical, and neonatal characteristics by BMI group.

Variable	Normal (*n* = 105)	Overweight (*n* = 145)	Obese I (*n* = 130)	Obese II (*n* = 75)	Obese III (*n* = 46)	*p*
Age (years), mean ± SD	27.4 ± 5.4	27.7 ± 5.4	27.5 ± 5.5	28.3 ± 5.3	28.5 ± 5.8	0.669
Height (cm), mean ± SD	163.1 ± 5.9	163.8 ± 5.8	164.0 ± 6.2	164.3 ± 5.7	163.2 ± 5.3	0.703
Weight (kg), mean ± SD	57.5 ± 5.8	73.3 ± 6.5	87.2 ± 7.5	100.9 ± 8.4	120.8 ± 11.6	<0.001
BMI (kg/m^2^), mean ± SD	21.6 ± 1.8	27.3 ± 1.4	32.4 ± 1.4	37.3 ± 1.4	45.3 ± 2.8	<0.001
Gestational age (weeks), mean ± SD	40.1 ± 1.3	40.1 ± 1.4	40.1 ± 1.5	40.2 ± 1.6	39.6 ± 1.8	0.374
Gravidity, mean ± SD	2.50 ± 1.27	2.41 ± 1.21	2.25 ± 1.33	2.23 ± 1.42	2.28 ± 1.31	0.209
Nulliparous, *n* (%)	36 (34.3)	46 (31.7)	60 (46.2)	36 (48.0)	20 (43.5)	0.043
Bishop score, median (IQR)	5 (4–6)	5 (3–6)	4 (3–6)	3 (2–4)	3 (2–4)	<0.001
Gestational weight gain (kg), mean ± SD	11.1 ± 1.6	8.5 ± 1.2	6.8 ± 1.2	5.7 ± 1.3	5.3 ± 1.2	<0.001
Birth weight (g), mean ± SD	3240 ± 437	3375 ± 383	3407 ± 443	3387 ± 381	3449 ± 349	0.019
Apgar 1 min, median (IQR)	9 (8,9)	8 (7–9)	8 (7–9)	8 (7–9)	8 (7–9)	0.016
Apgar 5 min, median (IQR)	10 (9,10)	9 (9,10)	9 (9,10)	9 (8–10)	9 (8–10)	0.058
Apgar 1 min < 7, *n* (%)	6 (5.7)	10 (6.9)	14 (10.8)	9 (12.0)	7 (15.2)	0.236
NICU admission, *n* (%)	3 (2.9)	5 (3.4)	7 (5.4)	6 (8.0)	4 (8.7)	0.338

Values are mean ± SD, median (IQR), or *n* (%) as appropriate. Kruskal–Wallis test for continuous/ordinal variables; chi-squared test for categorical variables. BMI: body mass index; IQR: interquartile range; NICU: neonatal intensive care unit; GWG: gestational weight gain (calculated as the difference between admission and self-reported pre-pregnancy weight).

**Table 2:** Delivery outcomes and Robson-stratified cesarean rates by BMI group.

BMI Group	Vaginal *n* (%)	Cesarean *n* (%)	*n*	Robson 2 *n*	R2 CS %	Robson 4 *n*	R4 CS %
Normal (18.5–24.9)	87 (82.9)	18 (17.1)	105	36	22.2	69	14.5
Overweight (25–29.9)	115 (79.3)	30 (20.7)	145	46	30.4	99	16.2
Obese I (30–34.9)	88 (67.7)	42 (32.3)	130	60	46.7	70	20.0
Obese II (35–39.9)	37 (49.3)	38 (50.7)	75	36	55.6	39	46.2
Obese III (≥40)	22 (47.8)	24 (52.2)	46	20	60.0	26	46.2
Total	349 (69.7)	152 (30.3)	501	198	41.4	303	23.1

CS: cesarean section; R2: Robson Group 2 (nulliparous, singleton, cephalic, term, induced); R4: Robson Group 4 (multiparous, no uterine scar, singleton, cephalic, term, induced). Cochran–Armitage trend test: overall Z = 6.099 (*p* < 0.001); Robson Group 2 Z = 3.688 (*p* < 0.001); Robson Group 4 Z = 4.448 (*p* < 0.001). Group 2 vs. Group 4 overall CS rate: χ^2^ = 18.14, *p* < 0.001.

**Table 3:** Cesarean delivery indications by BMI group.

Indication	Normal (*n* = 105)	Overweight (*n* = 145)	Obese I (*n* = 130)	Obese II (*n* = 75)	Obese III (*n* = 46)
Non-reassuring FHR, *n* (% of CS)	8 (44.4)	11 (36.7)	16 (38.1)	11 (28.9)	7 (29.2)
Prolonged active phase, *n* (% of CS)	4 (22.2)	8 (26.7)	8 (19.0)	8 (21.1)	4 (16.7)
Failed induction, *n* (% of CS)	3 (16.7)	4 (13.3)	9 (21.4)	8 (21.1)	5 (20.8)
CPD, *n* (% of CS)	2 (11.1)	3 (10.0)	5 (11.9)	8 (21.1)	4 (16.7)
Other, *n* (% of CS)	1 (5.6)	4 (13.3)	4 (9.5)	3 (7.9)	4 (16.7)
Failed induction/all women, *n* (%)	3 (2.9)	4 (2.8)	9 (6.9)	8 (10.7)	7 (15.2)

Rows 1–5: *n* (%) of cesarean deliveries within each BMI group (denominator = CS cases per group). Row 6 (failed induction/all women): denominator = all women in that BMI group, reflecting the overall failed induction rate. Overall distribution of indication types (rows 1–5) across BMI groups: χ^2^ = 6.56, df = 16, *p* = 0.981; because 11/25 cells had expected counts < 5, a Monte Carlo simulation (20,000 permutations) was also performed and yielded a concordant *p* = 0.982. The Cochran–Armitage trend test for failed induction (row 6) across ordered BMI categories: Z = 3.570, *p* < 0.001. Percentages may not total exactly 100% due to rounding. FHR: fetal heart rate; CPD: cephalopelvic disproportion.

**Table 4:** Induction duration (**A**), induction agents (**B**), and epidural analgesia (**C**) by BMI group.

Parameter	Normal (*n* = 105)	Overweight (*n* = 145)	Obese I (*n* = 130)	Obese II (*n* = 75)	Obese III (*n* = 46)
(**A**) Induction duration					
Total duration (h), mean ± SD	6.7 ± 3.1	7.3 ± 3.5	9.1 ± 4.1	10.1 ± 4.2	11.2 ± 4.6
Latent phase (h), mean ± SD	3.5 ± 2.1	3.8 ± 2.3	5.0 ± 2.8	5.5 ± 3.0	6.0 ± 3.1
Active phase (h), mean ± SD	3.2 ± 1.2	3.5 ± 1.4	4.1 ± 1.5	4.6 ± 1.4	5.2 ± 1.6
(**B**) Induction agents					
Oxytocin alone, *n* (%)	71 (67.6)	90 (62.1)	71 (54.6)	35 (46.7)	18 (39.1)
Foley + oxytocin, *n* (%)	21 (20.0)	27 (18.6)	31 (23.8)	21 (28.0)	13 (28.3)
Foley alone, *n* (%)	10 (9.5)	18 (12.4)	13 (10.0)	10 (13.3)	11 (23.9)
Dinoprostone, *n* (%)	3 (2.9)	10 (6.9)	15 (11.5)	9 (12.0)	4 (8.7)
Cervical ripening required, *n* (%)	34 (32.4)	55 (37.9)	59 (45.4)	40 (53.3)	28 (60.9)
(**C**) Epidural analgesia					
Epidural use, *n* (%)	5 (4.8)	8 (5.5)	9 (6.9)	5 (6.7)	4 (8.7)
Robson Group 2, *n* (% of R2)	3 (8.3)	5 (10.9)	6 (10.0)	4 (11.1)	3 (15.0)
Robson Group 4, *n* (% of R4)	2 (2.9)	3 (3.0)	3 (4.3)	1 (2.6)	1 (3.8)

Values are mean ± SD or *n* (%). (**A**) induction duration; (**B**) induction agents; (**C**) epidural analgesia. Kruskal–Wallis: total H = 62.28 (*p* < 0.001), latent H = 51.49 (*p* < 0.001), active H = 80.55 (*p* < 0.001). Cervical ripening χ^2^ = 15.90 (*p* = 0.003). Epidural χ^2^ = 1.13 (*p* = 0.890) across BMI groups; nulliparous vs. multiparous χ^2^ = 9.79 (*p* = 0.002). R2: Robson Group 2; R4: Robson Group 4.

**Table 5:** Multivariable logistic regression: independent predictors of cesarean delivery ((**A**) primary analysis using admission BMI; (**B**) sensitivity analysis using self-reported pre-pregnancy BMI).

Variable	aOR	95% CI	*p*-Value
(**A**) **Primary Analysis: Admission BMI as Exposure Variable.**			
BMI category (per one-level increase)	1.489	1.254–1.767	<0.001
Nulliparity	1.481	0.940–2.332	0.090
Admission Bishop score (per unit increase)	0.807	0.718–0.906	<0.001
(**B**) **Sensitivity analysis: self-reported pre-pregnancy BMI as exposure variable.**			
Pre-pregnancy BMI category (per one-level increase)	1.461	1.227–1.739	<0.001
Nulliparity	1.442	0.918–2.264	0.112
Admission Bishop score (per unit increase)	0.801	0.714–0.899	<0.001

aOR: adjusted odds ratio; CI: confidence interval. Reference categories: normal BMI for BMI category; multiparous for parity. Nulliparity was retained a priori regardless of statistical significance. (**A**) (admission BMI as exposure): Nagelkerke R^2^ = 0.174; Hosmer–Lemeshow goodness-of-fit test χ^2^ = 6.14, df = 8, *p* = 0.632, indicating adequate calibration; sensitivity analysis additionally including induction indication as covariate did not alter estimates (likelihood ratio χ^2^ = 2.24, *p* = 0.896). (**B**) (pre-pregnancy BMI as exposure): model AUC = 0.711; Cochran–Armitage trend test for cesarean delivery across pre-pregnancy BMI categories Z = 5.842, *p* < 0.001; eleven percent of women (*n* = 55) were classified into a different (one category higher) BMI stratum compared with admission BMI, reflecting the typical 2–3 kg/m^2^ shift introduced by gestational weight gain.

**Table 6:** Apparent and bootstrap optimism-corrected AUC values for prediction of cesarean delivery.

Model	Apparent AUC (95% CI)	Optimism	Optimism-Corrected AUC	DeLong vs. Bishop Alone
BMI alone	0.678 (0.625–0.730)	−0.001	0.679	Z = 0.37, *p* = 0.713
Bishop score alone	0.672 (0.620–0.721)	−0.000	0.672	Reference
Combined (BMI + Bishop + nulliparity)	0.715 (0.666–0.767)	+0.006	0.709	Z = 2.593, *p* = 0.010

AUC: area under the ROC curve; CI: confidence interval (bootstrap percentile, 2000 resamples). Optimism-corrected AUC was estimated using Harrell’s bootstrap method with 2000 resamples. Negative optimism values indicate that the bootstrap-corrected estimate is marginally higher than apparent AUC, which may occur with simple single-predictor models and small optimism magnitudes. DeLong test statistics compare each model to Bishop score alone (reference).

## Data Availability

The data presented in this study are available on request from the corresponding author. The data are not publicly available due to institutional policies protecting patient privacy.

## Abbreviations

The following abbreviations are used in this manuscript: aOR, adjusted odds ratio; ACOG, American College of Obstetricians and Gynecologists; AUC, area under the receiver operating characteristic curve; BMI, body mass index; CI, confidence interval; CPD, cephalopelvic disproportion; CS, cesarean section; FHR, fetal heart rate; GWG, gestational weight gain; IOM, Institute of Medicine; IQR, interquartile range; NICU, neonatal intensive care unit; OECD, Organisation for Economic Co-operation and Development; OR, odds ratio; ROC, receiver operating characteristic; SD, standard deviation; STROBE, Strengthening the Reporting of Observational Studies in Epidemiology; WHO, World Health Organization.
